# Combined Inhibition of IAPs and WEE1 Enhances TNFα- and Radiation-Induced Cell Death in Head and Neck Squamous Carcinoma

**DOI:** 10.3390/cancers15041029

**Published:** 2023-02-06

**Authors:** Tiffany Toni, Ramya Viswanathan, Yvette Robbins, Sreenivasulu Gunti, Xinping Yang, Angel Huynh, Hui Cheng, Anastasia L. Sowers, James B. Mitchell, Clint T. Allen, Ethan L. Morgan, Carter Van Waes

**Affiliations:** 1 Tumor Biology Section, Head and Neck Surgery Branch, National Institute on Deafness and Other Communication Disorders, National Institutes of Health, Bethesda, MD 20892, USA; 2 Pritzker School of Medicine, University of Chicago, Chicago, IL 60637, USA; 3 Section on Translational Tumor Immunology, National Institute on Deafness and Other Communication Disorders, National Institutes of Health, Building 10, Room 7N240C, Bethesda, MD 20892, USA; 4 Sinonasal and Skull Base Tumor Program, National Institute on Deafness and Other Communication Disorders, National Institutes of Health, Bethesda, MD 20892, USA; 5 Radiation Biology Branch, Center for Cancer Research, National Cancer Institute, National Institutes of Health, Bethesda, MD 20892, USA; 6 School of Life Sciences, University of Sussex, Brighton BN1 9QG, UK

**Keywords:** HNSCC, WEE1 inhibitor, cIAP inhibitor, tumor necrosis factor (TNF)

## Abstract

**Simple Summary:**

Both IAP and WEE1 inhibitors have demonstrated therapeutic efficacy in in vitro pre-clinical models of head and neck squamous cell carcinoma (HNSCC). Here, we demonstrate that dual treatment with IAP and WEE1 inhibitors sensitizes both HPV-negative and HPV-positive HNSCC cells to both TNFα-dependent and radiation-associated cell death, demonstrating a potential therapeutic combination to treat these cancers.

**Abstract:**

Head and neck squamous cell carcinoma (HNSCC) remains a prevalent diagnosis with current treatment options that include radiotherapy and immune-mediated therapies, in which tumor necrosis factor-α (TNFα) is a key mediator of cytotoxicity. However, HNSCC and other cancers often display TNFα resistance due to activation of the canonical IKK–NFκB/RELA pathway, which is activated by, and induces expression of, cellular inhibitors of apoptosis proteins (cIAPs). Our previous studies have demonstrated that the IAP inhibitor birinapant sensitized HNSCC to TNFα-dependent cell death in vitro and radiotherapy in vivo. Furthermore, we recently demonstrated that the inhibition of the G2/M checkpoint kinase WEE1 also sensitized HNSCC cells to TNFα-dependent cell death, due to the inhibition of the pro-survival IKK-NFκB/RELA complex. Given these observations, we hypothesized that dual-antagonist therapy targeting both IAP and WEE1 proteins may have the potential to synergistically sensitize HNSCC to TNFα-dependent cell death. Using the IAP inhibitor birinapant and the WEE1 inhibitor AZD1775, we show that combination treatment reduced cell viability, proliferation and survival when compared with individual treatment. Furthermore, combination treatment enhanced the sensitivity of HNSCC cells to TNFα-induced cytotoxicity via the induction of apoptosis and DNA damage. Additionally, birinapant and AZD1775 combination treatment decreased cell proliferation and survival in combination with radiotherapy, a critical source of TNFα. These results support further investigation of IAP and WEE1 inhibitor combinations in preclinical and clinical studies in HNSCC.

## 1. Introduction

Head and neck squamous cell carcinoma (HNSCC) is the sixth most common cancer, with over 600,000 new cases worldwide [1]. Prognosis varies significantly with cancer site, stage, and human papillomavirus (HPV) status [2], with the unadjusted five-year survival rate ranging between 28.8% and 58.7% for HPV-negative and 52.2% and 77.6% for HPV-positive HNSCC, depending on the tumor subsite [3]. Currently, limited treatment options for recurrent, metastatic HNSCC exist. New treatment options that sensitize HPV-negative HNSCC to radio- or chemoradiotherapy are especially needed to improve chances of survival. In comparison, HPV-positive HNSCC tends to affect a younger population and responds more favorably to radiotherapy, with novel treatment regimens aimed at de-escalating treatment-associated toxicities [4].

TNFα is a cytokine that is secreted by leukocytes, stromal, and cancer cells into the tumor microenvironment and may be induced by both immune and radiation therapies [5,6]. In turn, TNFα can promote apoptotic or necroptotic cell death through a variety of mechanisms. These include the extrinsic TNFα-receptor and Fas-associated death domain (TNFR-FADD) apoptosis or necroptosis pathways, an intrinsic pathway involving mitochondrial-initiated caspase activation, or via the induction of Janus Kinase (JNK) and reactive oxygen species (ROS), which can induce DNA damage [7]. These anti-cancer effects of TNFα may be inhibited by cellular inhibitor of apoptosis proteins (cIAPs), that can inhibit cell death pathway components, as well as promote the activation of transcription factor Nuclear Factor-kappaB (NFκB), that can further induce the expression of IAPs and other pro-survival proteins. Specifically, following TNFR activation, cIAP-mediated ubiquitination of RIP1 can promote the recruitment of the canonical inhibitor of nuclear factor-κB (IκB) kinase (IKK) complex and downstream NFκB/RELA (also known as p65)-dependent transcription of Baculoviral IAP repeat-containing protein (*BIRC) 2/3* encoding IAP1/2 and *BCL2* genes that inhibit caspase and mitochondrial mediated cell death pathways [8,9,10]. HNSCC frequently harbors genomic alterations in components of the TNFα-FADD cell death pathways and *BIRC* genes encoding cIAPs, enabling the evasion of TNFα-induced cytotoxicity [11,12]. cIAP inhibition has also been reported to inhibit RIP1 polyubiquitination and canonical IKK-NFκB/RELA prosurvival signaling, while promoting the stabilization of NFκB-inducing kinase (NIK) and non-canonical NFκB2/RELB transcription of TNFα [13,14], but these effects have not been demonstrated in HNSCC.

Birinapant, a second mitochondria-derived activator of caspase (SMAC) mimetic, is a validated small molecule inhibitor of cIAP1, an important inhibitor of caspase-mediated apoptotic and necroptotic cell death [15]. Our lab has shown that birinapant is effective in sensitizing HPV-negative HNSCC to TNFα in vitro, and radiation-induced cytotoxicity in vivo, especially those that harbor FADD/BIRC2 amplifications [16]. cIAP antagonist ASTX660 (also known as tolinapant) showed similar effects in HPV-negative as well as HPV-positive HNSCC, with increased levels of cell death occurring through apoptotic and necroptotic pathways [17,18,19]. Recently, another IAP inhibitor, Debio 1143 (xevinapant), demonstrated significant improvements in overall survival, progression-free survival, and the locoregional control rate at 18 months when combined with chemoradiotherapy in a phase II clinical trial for local regionally advanced HPV-negative HNSCC [20]. Following these promising results, Debio 1143 was granted breakthrough therapy designation from the FDA and an international phase III clinical trial, TrilynX, investigating Debio 1143 in combination with chemoradiotherapy remains underway [21,22,23,24].

WEE1 cell cycle inhibitors are another class of novel small molecule therapeutic inhibitors, that are important regulators of the DNA damage response [25]. WEE1 kinase is a G2/M cell cycle checkpoint protein that enables DNA damage repair prior to mitotic entry. Rapidly proliferating cancer cells exhibit genetic instability and amass cumulative DNA damage that requires WEE1 and an intact checkpoint for adequate repair. WEE1 inhibitors can promote cell death of genomically unstable cancers as individual agents, and also have the potential to synergize with radiotherapy and other DNA-damaging chemotherapies by limiting S/G2/M phase checkpoint repair, enhancing chromosomal damage and mitotic catastrophe [26]. AZD1775 (also known as adavosertib) represents a potent WEE1 inhibitor under investigation in a variety of cancer types in combination with chemoradiotherapy [27,28,29].

We recently described a novel interaction between WEE1 and the TNFα-IKK-NFκB prosurvival pathway that provides a possible rationale for combination therapy of WEE1 with cIAP inhibitors to enhance TNFα-induced cell death [30]. In particular, TNFα activation of the IKKa/b complex enhances activation of WEE1 and CDC2, to promote G2/M pause while enhancing TNFα-NFκB prosurvival signaling. Conversely, inhibiting WEE1 enhances DNA damage and decreases downstream pro-survival NFκB signaling and expression of BCL2, favoring cell death. Individual agents, both AZD1775 and birinapant have shown favorable results when combined with TNFα and radiotherapy in preclinical murine studies [16,30]. Here, we explore the anticancer effects of combination treatment with WEE1 and cIAP inhibitors with TNFα and radiotherapy, and characterize associated changes in cell death and DNA damage to gain mechanistic insights into the observed effects.

## 2. Materials and Methods

### 2.1. Therapeutic Reagents

AZD1775 (adavosertib), birinapant (TL32711), and ASTX660 (tolinapant) were purchased from MedChemExpress at a stock concentration of 10 mM dissolved in dimethyl sulfoxide (DMSO). Tumor necrosis factor α with carrier (TNFα) was purchased from Biotechne R&D Systems and dissolved in 1% Bovine Serum Albumin in PBS at a stock concentration of 40 μg/mL.

### 2.2. HNSCC Cell Lines

A panel of genotype validated and sequenced HPV positive and negative HNSCC cell lines were obtained from Dr. T.E. Carey from the University of Michigan [31]. One additional HPV-positive cell line (UPCI:SCC090) was obtained from Dr. Susanne M. Gollin from the University of Pittsburgh. HPV-negative cell lines were cultured in minimal essential medium (MEM) supplemented with 10% fetal calf serum, penicillin and streptomycin (100 µg/mL), and L-glutamine. HPV-positive cell lines were cultured in Dulbecco’s minimal essential medium (DMEM) with the same additives. All cancer cell lines were cultured for no more than 20 passages. Human primary oral keratinocytes (HOK) from oral gingival mucosa were purchased from Science Cell Research laboratories and used as a control cell line and cultured in serum-free oral keratinocyte medium with supplements (Science Cell) for fewer than 6 passages. Cells were incubated at 37 °C with 5% CO_2_ under humidified conditions and preserved in freezing media containing DMSO for long-term storage.

One additional TP53 knockout (KO) UMSCC74A cell line was generated using CRISPR-Cas9. CRISPER-CAS9 based UMSCC74_P53KO cell pool was generated by Synthego using the guide RNA 5′ CCAUUGCUUGGGACGGCAAG 3′. The knockout efficiency (96% INDEL efficiency) of the Crisper edited pool was determined by the sequencing of genomic DNA using the primers forward 5′CAGGCATTGAAGTCTCATGGAAG3′ and reverse 5′ ACCTATGGAAACTGTGAGTGGATC3′.

### 2.3. siRNA Depletion

UMSCC-1 or UMSCC-47 cells were plated 2 × 10^5^/well on 6-well plate and transfected overnight with siRNA targeting WEE1 (si21 and si23; Thermo Fisher Scientific, Cat#4392420) or negative control siRNA (Dharmacon, Cat#D-001810-0X) at a final concentration of 10 nM using Lipofectamine RNAiMax (Life Technologies, Cat#13778150) in MEM media containing no antibiotics. The medium was changed to the normal culture media the following morning and experimental therapeutics added 8 h later.

### 2.4. Western Blot

Following drug treatments or siRNA transfection, cells were washed with 1X PBS, and disadhered with 0.25% trypsin prior to lysis in SDS-lysis buffer (50 mM Tris pH 8.0, 100 mM NaCl, 1% SDS, and 10 mM EDTA) and protease and phosphatase inhibitor cocktail (HALT protease and phosphatase inhibitor cocktail, Thermo Scientific, Waltham, MA, USA). The Pierce BCA Protein Assay Kit (Thermo Scientific) was used to quantify the protein concentration of each sample. Lysates were run on NuPAGE 4–12% gradient Bis-Tris gels (Invitrogen) and transferred to nitrocellulose membranes using the Invitrogen iBlot 2 system, according to the manufacturer’s standard protocol. After blocking for 1 h in Odyssey blocking buffer (Li-COR Biosciences, Lincoln, NE, USA), membranes were incubated with the primary antibodies overnight at 4 °C. After washing in TBS buffer with added Tween, membranes were incubated with species-specific IR dye-conjugated secondary antibodies for 1 h at room temperature. The signal was visualized using LI-COR ODYSSEY CLx Infrared Imaging System (Li-COR Biosciences). The following antibodies were used at 1:1000 dilution in Odyssey antibody diluent (Li-COR Biosciences, Lincoln, NE, USA) unless otherwise stated: WEE1 (1:500; sc-5285, Santa Cruz Biotechnology (SCBT)), pCDC2 (Y15; 8242, Cell Signaling Technology (CST)), CDC2 (9116, CST), pIKKα/β (S176/S177—1:500; 2078, CST), IKKα (11930, CST), IKKβ (8943, CST), pRELA (S536; 3033, CST), RELA (3039, CST), BCL2 (15071, CST), PARP (9532, CST), Cleaved Caspase 3 (N175—1:500; 9664, CST), Caspase 3 (14220, CST), γ-H2AX (9718, CST), phospho-Histone H3 (S10; 53348, CST), Histone H3 (4499, CST), β-actin (1:2000, ab8226, Abcam).

### 2.5. Real-Time Impedance Assay

Cells were plated at 5000 cells per well on a 96-well E-plate (ACEA Biosciences, San Diego, CA, USA). After overnight adherence, cells were cultured with the indicated experimental therapeutic with or without TNFα. Changes in cell density by impedance were acquired using the xCELLigence Real-Time Cell Analysis (RTCA) platform according to the manufacturer’s protocol (ACEA Biosciences). Measured electrical impedance is translated as a dimensionless parameter, the Cell Index (CI), which was normalized to the 0.01% DMSO control. A blank reading prior to the addition of cells is taken to zero the system. Higher CI values are associated with increased cell density that increases resistance to electrical currents. All impedance assay conditions were performed with six replicates.

### 2.6. Colony Formation Assays

Cells were treated with the drugs indicated at the concentrations indicated. 0.01% DMSO-treated cells were used as a control. At 24 h post-treatment, cells were trypsinised and reseeded in 6-well plates at 500 cells per well and left to form colonies for 10–14 days. Colonies were then stained (1% crystal violet, 25% methanol) and were counted manually. Each condition was performed in triplicate. Images of the wells were obtained using EVOS brightfield scanning.

### 2.7. Cell Cycle Analysis

Cells were plated in 6-well dishes at 150,000 cells/well and treated with the indicated drugs the following day. As a control, 0.01% DMSO-treated cells were used. At the indicated time point, the supernatant and trypsinized cells were collected by centrifugation and processed by following the protocol provided by Cycletest Plus DNA Reagent Kit (BD Biosciences, San Jose, CA, USA) prior to analyzing on a FACS Fortessa flow cytometer (BD Biosciences). Each condition was performed in triplicate and data from 10,000 cells per sample were analyzed using Flow-Jo analysis software (Tree Star).

### 2.8. Flow Cytometry Analysis

Cells were plated in 6-well dishes at 150,000 cells/well and treated with the indicated drugs the following day. As a control, 0.01% DMSO-treated cells were used. At the indicated time point, the supernatant and trypsinized cells were collected by centrifugation and fixed in 4% paraformaldehyde at RT for 15 min. Cells were then washed in PBS and permeabilized in 90% methanol. Before the addition of antibodies, cells were blocked in 5% BSA in PBS with 0.1% Triton X-100. Cells we then incubated with a PE conjugated γ-H2AX antibody (1:100; 5763, CST) prior to analyzing on a FACS Fortessa flow cytometer (BD Biosciences). Each condition was performed in triplicate and data from 10,000 cells per sample were analyzed using Flow-Jo analysis software (Tree Star).

### 2.9. Annexin V Assay

Cells were plated in 6-well dishes at 150,000 cells/well and treated with the indicated drugs the following day. As a control, 0.01% DMSO-treated cells were used. At the indicated time point, the supernatant and trypsinized cells were collected by centrifugation. For HPV-negative samples, Annexin V apoptosis assay (TACS Annexin V kit; 4830-250-K) was performed as indicated on the product datasheet. For HPV-positive cell lines, cells were washed in PBS and were then incubated with an Alexa Fluor^®^ 488 conjugated phosphatidylserine (PS) antibody (1:100; 16–256, Sigma-Aldrich). Cells were then stained with propidium iodide (PI) for 30 min and then fixed in 4% paraformaldehyde for 15 min. Cells were then analyzed on a FACS Fortessa flow cytometer (BD Biosciences). Each condition was performed in triplicate, and data from 10,000 cells per sample were analyzed using Flow-Jo analysis software (Tree Star). Early apoptotic cells were defined as Annexin positive/PI negative and late apoptotic cells was defined as Annexin-positive/PI-positive.

### 2.10. XTT Viability and Cell Death Assay

Cells were plated in the inner 60 wells of 96-well plates at 5000 cells per well. The next day, cells were treated with the indicated experimental therapeutic at the concentration indicated. After 72 h, XTT reagent was added per the manufacturer’s instructions and plates were read at 450 nm on a plate reader. Experimental background readings taken at 660 nm were subtracted from experimental readings and then normalized to DMSO control readings. For cell death analysis, 1X EmbryoMax nucleosides (Millipore Sigma, Burlington, MA, USA), 20 μM ZVAD (BD Biosciences), and/or 20 μM necrostatin (BD Biosciences) were added at the same time as the experimental drugs.

### 2.11. In Vitro Radiation

Cells were exposed to experimental drugs for 1 h prior to single-dose 4 Gy radiation for UMSCC-47 cells and 6 Gy radiation for UMSCC-1 cells. For the DNA damage time course study, cells were pretreated with experimental drugs for 24 h.

Cells were irradiated with separate XRAD320 X-ray irradiators (Precision X-ray, Inc., North Branford, CT, USA) housed in the Radiation Biology Branch of the National Cancer Institute. For all experiments, ionizing radiation was delivered at a dose rate of ~2.42 Gy/min with 300 kV X-rays at a distance of ~50 cm from the radiation source.

### 2.12. NFκB Reporter Analysis

The stable reporter line, UMSCC-1κB, established by transfecting UMSCC-1 cells with a pLenti-based vector containing 6 repeated κB-binding sites upstream of a b-lactamase reporter gene (pLenti-bsd-NFκB-bla; created using pLenti6/V5-DEST Gateway vector (V49610; Life Technologies, Carlsbad, CA, USA), a NFκB response element sequence, and a blasticidin resistance gene for selection [30]. The β-lactamase reporter enzyme can cleave a fluorescent FRET substrate (LiveBLAzer FRET-B/G Loading Kit with CCF4-AM; Life Technologies, cat. #K1095), which disrupts FRET and results in blue fluorescence. The blue:green fluorescence ratio thus indicates the activity of the NFκB reporter: cell viability.

### 2.13. qRT-PCR Analysis

Cells were treated as indicated in the figure legends. Total RNA was isolated using Trizol and RNeasy Mini Kit (Qiagen, Hilden, Germany) combined method per manufacturer’s protocol. cDNAs were synthesized using the High-Capacity cDNA Reverse Transcription Kit (Life Technologies) and qRT-PCR was performed on a QuantStudio 6 Flex Real-Time PCR system (Applied biosystems/Thermo Fisher Scientific). Predesigned Taqman primer/probe set were purchased from Life Technologies. Relative gene expression was normalized to GAPDH (Thermo Fisher Scientific, Cat#:4331182: GAPDH—hs99999905_m1) as an internal control, and fold changes were adjusted to the control samples. The cells in each experiment were transfected in duplicates, and each sample was assayed by qRT-PCR in triplicates. 2^−ΔΔCt^ was calculated and used as an indication of the relative expression levels. Data were presented as mean ± standard deviation (SD) from triplicates, and statistical analyses were performed using two-tailed, unpaired Student’s *t*-test.

### 2.14. ELISA

Cells were treated as indicated in the figure legends. The human TNFα DuoSet® ELISA was purchased from R&D Systems (DY210-05) and was used according to the manufacturer’s instructions.

### 2.15. Statistical Analysis

Cell viability was analyzed for synergy using CompuSyn (ComboSyn Inc., Paramus, NJ, USA) and Bliss scores were calculated using SynergyFinder (Netphar, University of Helsinki, Helsinki, Finland). All graphs were prepared using the GraphPad Prism (GraphPad, San Diego, CA, USA). Error bars represent mean ± the standard deviation. Statistical significance was determined as follows: NS = not significant, * *p* < 0.05, ** *p* < 0.01, *** *p* < 0.001.

## 3. Results

### 3.1. Combined Inhibition of IAPs and WEE1 Enhances the Inhibitory Effects of TNFα on Proliferation and Survival of HNSCC In Vitro

**Figure 1:**
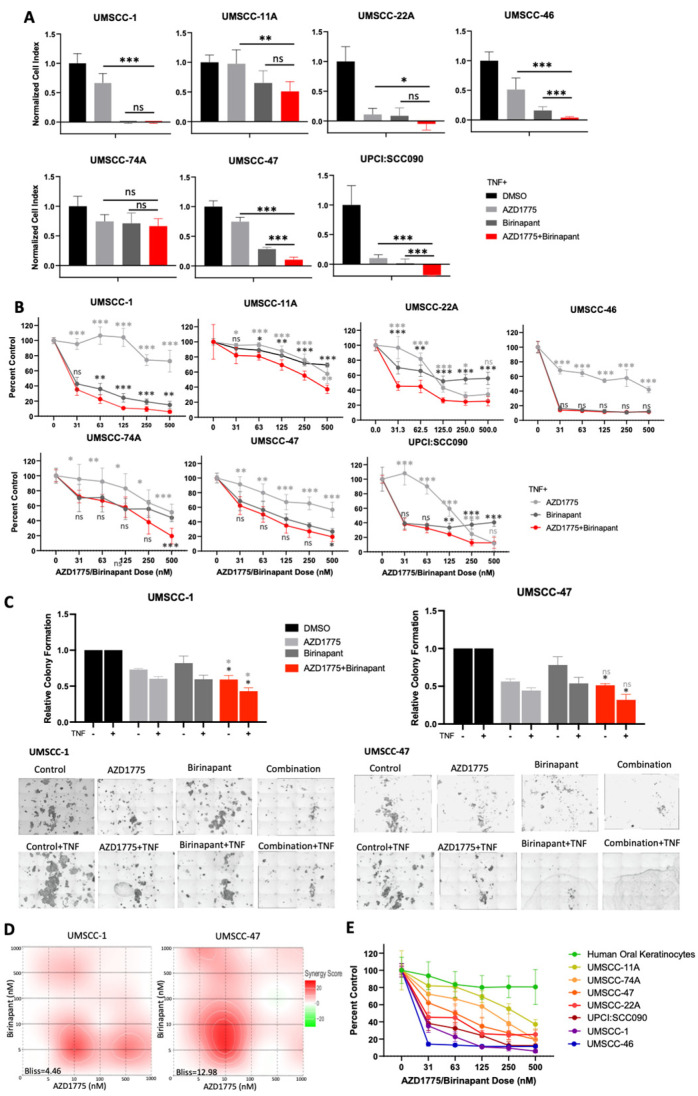
AZD1775 and birinapant combination treatment has therapeutic efficacy in HPV+ and HPV- HNSCC cells in the presence of TNF. (**A**) Impedance assay and (**B**) XTT viability analysis at 72 hrs after drug treatment, normalized to TNF control. AZD1775 and birinapant 100 nM with the exception of UMSCC-46, which has 2 nM birinapant and 500 nM AZD1775; TNF = 20 ng/mL, n = 6. Single outlier excluded from UMSCC-11A 500 nM AZD1775 group in XTT analysis. (**C**) Colony formation assay in UMSCC-1 and UMSCC-47. AZD1775 = 500 nM, birinapant = 500 nM, TNF = 20 ng/mL. n = 3. (**D**) Bliss Synergy scores according calculated using Synergyfinder software for AZD1775 and birinapant dose responses (0, 5, 10, 100, 500, 1000 nM) and TNF = 20 ng/mL. (**E**) XTT viability analysis with HOK cells compared to a panel of HNSCC cell lines; TNF = 20 ng/mL, n = 6. ns = not significant; * *p* < 0.05; ** *p* < 0.01; *** *p* < 0.001.

In order to investigate potential synergy between IAP and WEE1 inhibition, the small molecule inhibitors birinapant and AZD1775 were applied alone and in combination across a panel of HNSCC cell lines and cell proliferation and viability were assessed. In the absence of TNFα, most cell lines were resistant to birinapant alone, and combination treatment demonstrated modest enhancement beyond single drug treatments in the majority of HNSCC cell lines investigated (Supplementary Figure S1A–C). To determine if these inhibitors sensitized cells to TNFα-induced toxicity, single- or combination-treated cells were supplemented with 20 ng/mL TNFα; the addition of TNFα to combination-treated cells further enhanced the reduction in proliferation and cell viability beyond the single drug controls across the majority of a panel of cell lines (Figure 1A,B). To confirm these data were not specific to birinapant and AZD1775 treatment, we used the other IAP inhibitor ASTX600, an IAP inhibitor that more broadly targets cIAP1, cIAP2, and XIAP and has demonstrated safety in a phase I clinical trial [32]. Similar reductions in cell viability were observed, which were TNFα dependent, particularly in UMSCC-47 (Supplementary Figure S2A,B). Of note, both cell lines responded better to birinapant, so we used this IAP inhibitor for subsequent experiments (Supplementary Figure S2B). Next, we investigated the effects of combination treatment, in the presence or absence of TNFα, in more detail in UMSCC-1, an HPV-negative HNSCC cell line, and UMSCC-47, an HPV-positive cell line. First, we confirmed that both birinapant and AZD1775 were active at the doses used for subsequent experiments. As expected, both compounds inhibited their respective targets (cIAP1 expression for birinapant and phosphorylated CDC2 for AZD1775) in both cell lines at 500 nM (Supplementary Figure S3A,B). Next, we looked at the long-term effects of combination treatment using colony formation assays. In both cell lines, combination treatment significantly reduced colony formation when compared with the individual drugs alone, and this was enhanced in the presence of TNFα (Figure 1C). In order to assess if the effects of combination treatment were synergistic in HNSCC cells, we performed viability assays after treatment with a range of drug combinations, with or without TNFα., and performed Bliss analysis [33]. UMSCC-1 and UMSCC-47 showed moderate drug synergism (Bliss score > 1) across a dose range of 0–1 μM with Bliss synergy scores of 4.46 and 12.98 in the presence of TNFα, respectively (Figure 1D). Additionally, combination treatment resulted in a dose-dependent reduction in cell viability in all HNSCC cell lines tested, which was enhanced in the presence of TNFα (Figure 1E and Supplementary Figure S1D). Importantly, combination treatment only modestly inhibited the viability of primary human oral keratinocytes, indicating that the combination is not as toxic to nonmalignant cells. Taken together, these results demonstrate the potential for combined IAP and WEE1 inhibition to exert anti-cancer activity and sensitize HNSCC cells to TNFα-induced cytotoxicity.

### 3.2. Combination Treatment Induces Cell Death through Multiple Cell Death Pathways

**Figure 2:**
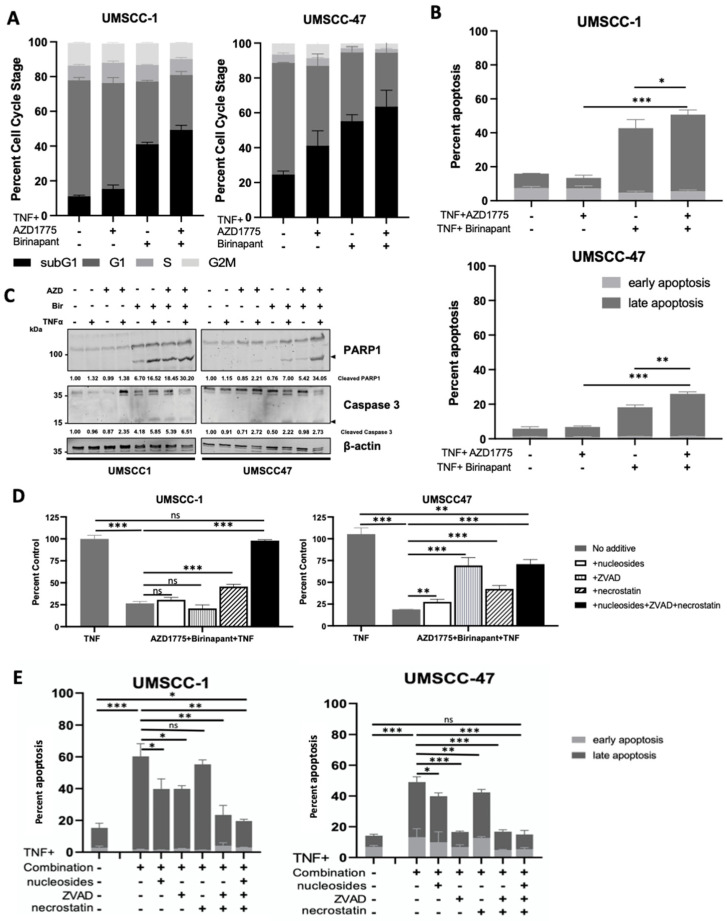
Combination treatment induces cell death in HNSCC through activation of multiple death pathways. (**A**) Cell cycle and (**B**) Annexin apoptosis assay analysis at 48 h after treatment. AZD1775/Birinapant = 100 nM, TNF = 20 ng/mL; n = 3. (**C**) Representative Western blot analysis of PARP and uncleaved/cleaved Caspase 3 expression following 48 h drug treatment. AZD1775 = 500 nM, birinapant = 500 nM, TNF = 20 ng/mL. β-actin was used as a loading control. (**D**) XTT viability assay at 72 h following simultaneous treatment with 500 nM AZD1775/Birinapant in the presence of 20 ng/mL TNF +/− 20 uM necrostatin, 20 uM Z-VAD-FMK, 1x nucleosides supplementation. n = 3. (**E**) Annexin apoptosis assay at 24 h following simultaneous treatment with 500 nM AZD1775/birinapant in the presence of 20 ng/mL TNF +/− 20 uM necrostatin, 20 uM Z-VAD-FMK, 1x nucleosides supplementation. n = 3. ns = not significant; * *p* < 0.05; ** *p* < 0.01; *** *p* < 0.001.

To investigate the effects of combination treatment further, we investigated the effects on the cell cycle as our previous results demonstrated that both birinapant and AZD1775 altered the cell cycle in HNSCC cells [16,30]. In the absence of TNFα, low-dose AZD1775 alone minimally increased sub-G1 DNA fragmentation of cells in UMSCC-1 compared with UMSCC47 cells. Conversely, only birinapant alone increased sub-G1 fraction in UMSCC-1 without TNFα (Supplementary Figure S4A). Combination treatment without TNFα did not significantly increase sub-G1 in either cell line compared with single drug treatments. With the addition of TNFα, combination treatment increased sub-G1 to a greater extent than the single drugs in both UMSCC-1 and UMSCC-47 cells at 48 h (Figure 2A). Sub-G1 representing fragmented DNA is a marker for cell death [34]. To confirm that the combination treatment was inducing cell death, we performed Annexin V assays to investigate apoptosis (Figure 2B and Supplementary Figure S4B). In UMSCC-1 and UMSCC-47 cells, external supplementation with TNFα resulted in significantly increased apoptosis in the combination-treated cells at 48 h. Furthermore, Western blot analysis demonstrated increased PARP and caspase 3 cleavage, indicating apoptotic cell death, and this was enhanced upon combination treatment in the presence of TNFα, particularly in UMSCC-47 cells (Figure 2C). To further investigate the mechanism of cell death occuring upon combination treatment, we performed both XTT and Annexin V assays and pre-treated cells with nucleosides (to enhance DNA repair and protect against mitotic catastrophe [35]), Z-VAD-FMK (ZVAD, a pan-caspase inhibitor), and/or Necrostatin-1 (a RIPK1 inhibitor, a critical component of the necroptotic cell death pathway). In UMSCC-1, pre-treatemt with ZVAD, nucleosides, and necrostatin alone had minor effects; however, supplementation with all three compunds completely restored cell viability. In contrast, all compounds indivdually partially restored cell viability in UMSCC-47 cells, with the greatest effects from ZVAD or all 3 compounds combined (Figure 2D,E and Supplementary Figure S4C,D). These data suggest that combined inhibition of IAP- and WEE1-associated cytotoxicity occurs via multiple mechanisms of cell death.

### 3.3. Depletion of WEE1 in Combination with IAP Inhibiton Also Enhances the Inhibitory Effects of TNFα on Proliferation and Survival of HNSCC In Vitro

**Figure 3:**
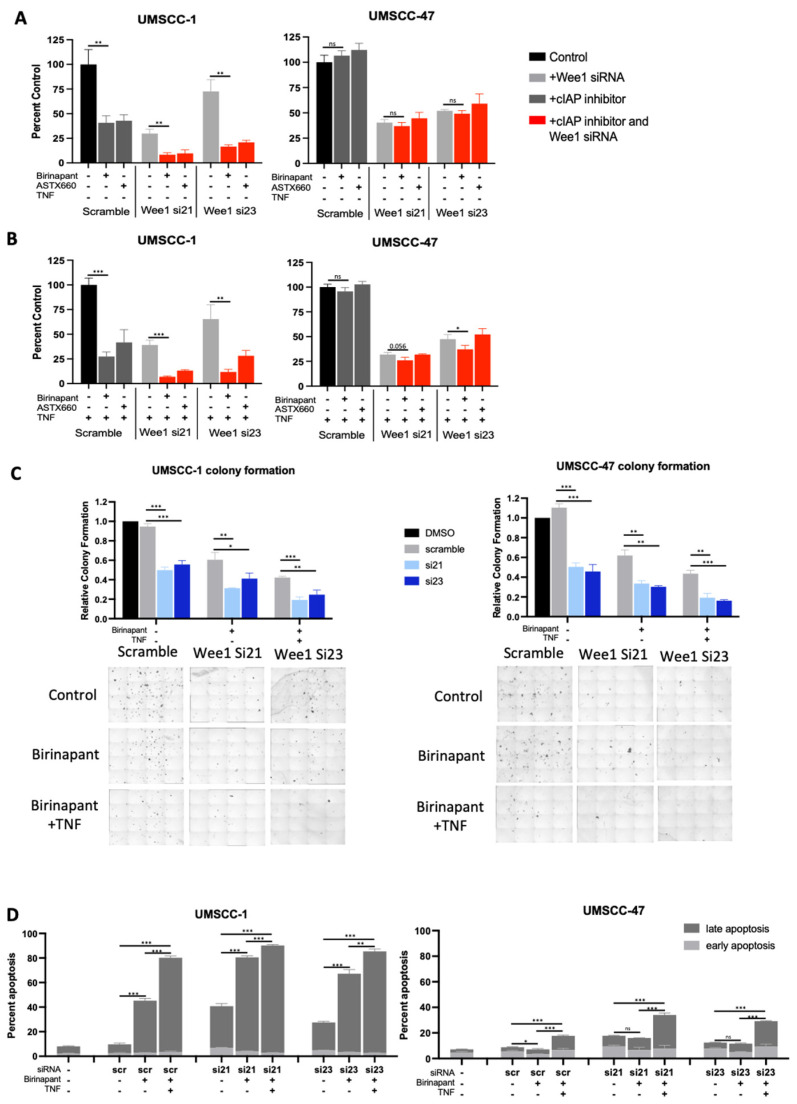
Alternative cIAP and WEE1 inhibition produce comparable effects in combination. XTT viability analysis of birinapant and ASTX660 in the presence of WEE1 siRNA (si21 and si23); AZD1775/ASTX660 = 500 nM, without TNF (**A**) and with TNF = 20 ng/mL (**B**). n = 3. (**C**) Colony formation data in UMSCC-1 and UMSCC-47. AZD1775 = 500 nM, birinapant = 500 nM, TNF = 20 ng/mL. (**D**) Annexin apoptosis assay at 24 h following simultaneous treatment with 500 nM birinpant, TNF= 20 ng/mL. ns = not significant; * *p* < 0.05; ** *p* < 0.01; *** *p* < 0.001.

Although AZD1775 is a well characterised WEE1 inhibitor, it has also been shown to inhibit WEE1 homologs and other G2/M kinases [36]. We therefore depleted WEE1 expression using two distinct siRNAs to determine if the combination effects observed were due to IAP and WEE1 activities. We confirmed WEE1 depeletion and a subsequent decrease in phosphorylated CDC2 in both UMSCC-1 and UMSCCC-47 after siRNA treatment (Supplementary Figure S3C). Next, cells treated with scramble siRNA or WEE1 siRNA were treated with birinapant or ASTX660, with or without TNFα. WEE1 depletion alone significanly reduced cell viability in both UMSCC-1 and UMSCC-47 cells and this was enhanced in the presence of TNFα, as we previously demonstrated [30]. The addition of either IAP inhibitor further reduced cell viability, which was again enhanced in the presence of TNFα (Figure 3A,B). These data were also confirmed used a colony formation assay, with the WEE1 siRNA/birinapant-combination-treated cells having a signifcant reduction in colony formation, which was further reducted in the presence of TNFα (Figure 3C). Finally, we confirmed that birinapant and WEE1 depeletion resulted in significant apoptosis upon TNFα addition, particularly in UMSCC-1 (Figure 3D). These results are consistent with the data produced using the WEE1 small molecule inhibitor, suggesting that the effects observed with AZD1775 are predominantly from WEE1 inhibition rather than off-target effects on other kinases.

### 3.4. Combination Treatment Modulates the IKK-NFκB in HNSCC Cells

A critical survival mechanism in the response to TNFα is the IKK-NFκB pathway [37]. Previous data has demonstrated that cIAP inhibitors can increase NFκB activity, whereas AZD1775 treatment suppresses NFκB activity [14,30]. As combined inhibition of IAP/WEE1 sensitised HNSCC cells to TNFα-cytotoxicity, we examine the effect of combination treatment on NFκB activity. We treated UMSCC-1 NFκB reporter cells [30] with each drug alone or in combination before treating cells with TNFα. As expected, TNFα-induced NFκB activity about 3-fold in the control cells (Supplementary Figure S5A). As we previously observed, AZD1775 significantly reduced TNFα-induced NFκB activity. Birinapant treatment significantly enhanced basal NFκB activity without effecting TNFα-induced NFκB activity, consistent with previous reports of IAP antagonists inducing NFκB activation [38,39]. Combination treatment reduced both birianpant-induced basal NFκB activity and TNFα-induced NFκB activity. To investigate the effects on the NFκB pathway in more detail, we looked at the activation of components of the canonical NFκB pathway by Western blot. As expected, TNFα treatment induced IKKα/β and RELA phosphorylation in control cells (Supplementary Figure S5B). Pretreatment with AZD1775 led to a slight reduction in both IKKα/β and RELA phosphorylation, consistent with our previous results [30]. In contrast, birinapant induced basal phosphorylation, but did not affect TNFα-induced IKKα/β and RELA phosphorylation, in line with the NFκB reporter assay data. However, combination treatment resulted in a much greater reduction in both IKKα/β and RELA phosphorylation. Together, these data suggest that combination treatment with IAP and WEE1 inhibitors results in a small but significant reduction in NFκB activity, and this may contribute to the increased sensitivity of TNFα-induced cyctotoxicity observed in HNSCC cells.

### 3.5. Combination Treatment Sensitizes HNSCC to Radiation Treatment and Induces DNA Damage

**Figure 4:**
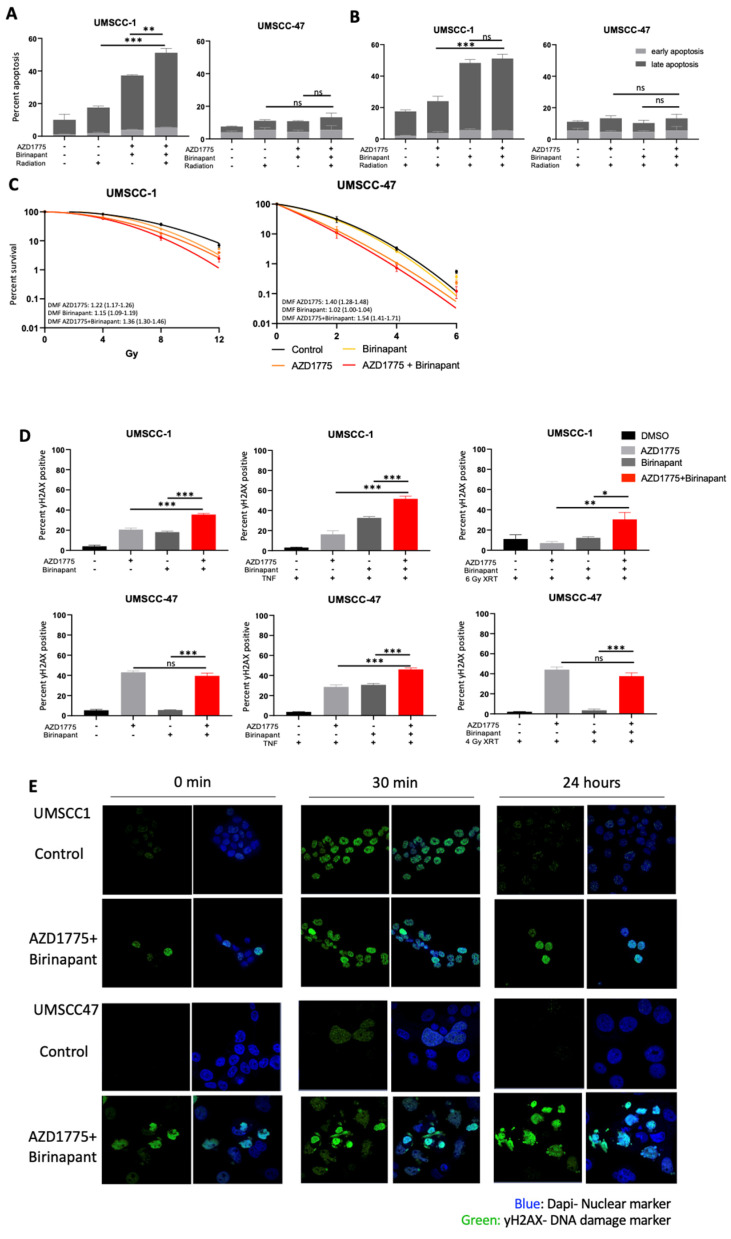
Combination treatment radiosensitizes HNSCC cells via the induction of sustained DNA damage. (**A**) Annexin apoptosis assay at 24 post single-dose radiation with post radiation with a 1 h pretreatment of AZD1775/Birinapant; XRT = 4 Gy for UMSCC-47 and 6 Gy for UMSCC1. AZD1775/Birinapant = 500 nM. (**B**) Annexin apoptosis assay at 24 post single-dose radiation with a 1 h pretreatment of AZD1775/Birinapant; XRT = 4 Gy for UMSCC-47 and 6 Gy for UMSCC1. AZD1775/Birinapant = 500 nM. (**C**) Clonogenic survival assay with DMF score following 24 h treatment with AZD1775/Birinapant at 500 nM compared with DMSO control. Single-dose ionizing radiation was administered 1 h following AZD1775/Birinapant pretreatment in UMSCC-1 (XRT = 0, 4, 8, 12 Gy) and UMSCC-47 (XRT = 0, 2, 4, 6 Gy). (**D**) Flow cytometry analysis of γ-H2AX positivity in single drug or combination-treated cells with or without 20 ng/mL TNF, or radiation. Radiation was delivered following a 1 h pretreatment at 6 Gy for UMSCC-1 and 4 Gy in UMSCC-47. AZD1775/Birinapant = 500 nM, n = 3. (**E**) Immunofluorescence analysis of γ-H2AX foci formation over a time following radiation (UMSCC-1 = 6 Gy, UMSCC-47 = 4 Gy) with a 24 h drug pretreatment. Cells were analysed 30 min and 24 h post radiation treatment. Time 0 represents unirradiated cells. AZD1775/Birinapant= 500 nM. ns = not significant; * *p* < 0.05; ** *p* < 0.01; *** *p* < 0.001.

As individual agents, both birinapant and AZD1775 have demonstrated the ability to sensitize HNSCC to radiation-induced cell death [16,30]. Radiation is also a potent stimulator of TNFα production in the tumor microenvironment, which we previously showed increases the effectiveness of birinapant and AZD1775 as individual agents [16,30]. To elucidate the possibility of combination treatment to sensitize cells to radiotherapy, UMSCC-1 and UMSCC-47 cells were treated with birinapant and/or AZD1775 for 1 h prior to single-dose ionizing radiation. A higher dose of 6 Gy was used in UMSCC-1, compared to 4 Gy in UMSCC-47, to account for increased intrinsic radioresistance in this HPV-negative HNSCC cell line as previously observed [40]. Twenty-four hours post treatment, radiation treatment alone induced apoptosis in UMSCC-1 cells; pretreatment with the drug combination significantly enhanced radiation-induced apoptosis (Figure 4A). This change appears to be primarily driven by birinapant-associated apoptosis following radiation exposure in short term apoptosis assay (Figure 4B). In UMSCC-47 cells, no significant level of apoptosis was observed upon radiation treatment, either with or without combination pretreatment in the short-term assay. The ability of the combination treatment to radio-sensitize HNSCC cells was further assessed by clonogenic survival assay. In both UMSCC-1 and UMSCC-47 cells, the individual drugs significantly increased the response to radiation treatment; however, the combination treatment significantly reduced survival compared with either drug alone in both cell lines (Figure 4C).

WEE1 is a critical componant of the DNA damage response and WEE1 inhibitors can synergize with other DNA damaging agents through accumulation mutagenesis and induction of mitotic catastrophe [26]. We therefore investigated if combination treatment could induce DNA damage in HNSCC cells. We first assessed the levels of γ-H2AX, a marker for DNA damage [41], by flow cytometry. In UMSCC-1, γ-H2AX expression increased following single drug treatment for 24 h; this was significantly increased upon combintion treatment (Figure 4D). In UMSCC-47, DNA damage was only induced by AZD1775 alone and the combination treatment. The addition of TNFα significantly enhanced the DNA damage induced by the combination in both cell lines; interestingly, TNFα addition also induced DNA damage in combination with birinapant in UMSCC-47. Radiation treatment also increased γ-H2AX expression after pretreatment with birinapant and AZD1775 in UMSCC-1, but not in UMSCC-47.

This may be due to the much lower levels of TNFα released by radiation treatment compared with TNFα supplementation [42,43]. Therefore, a higher dose of ionizing radiation or a longer time course may be required to see the effects of birinapant-associated DNA damage and a resultant combinatorial effect in UMSCC-47.

To analyse the response to radiation and the effects on DNA damage further, we performed confocal microscopy to look at the formation γ-H2AX foci. In the absense of radiation, combination-treated cells express higher levels of γ-H2AX foci, with many cells also demonstrating pan-nuclear staining, abnormal nuclear shape/architecture, aberrant mitoses, and apoptotic bodies compared to control cells in both cell lines (Figure 4E). 30 min post-radiation treatment, γ-H2AX foci levels were similar between control and combination treatment in UMSCC-1 cells; however, γ-H2AX foci levels were higher in the combination treatment in UMSCC-47 cells. By 24 h, γ-H2AX foci had resolved in both cells lines in the control; in combination-treated cells, however, γ-H2AX foci remained high, suggesting a sustained defect in DNA damage repair. Structural changes in UMSCC-47 nuclei are particularly apparent, which is consistent with the increased sensitivity of this cell line to radiotherapy compared to UMSCC-1 [40,44,45]. Taken together, these data suggest that combined inhibiton of IAP and WEE1 sensitizes HNSCC to radiation treatment. Furthermore, combination treatment induces DNA damage in combination with TNFα and radaition and may lead to a defect in DNA damage repair.

### 3.6. Mutated TP53 Is a Potential Determinant of Sensitivity to Combination Therapy in HNSCC Cells

**Figure 5:**
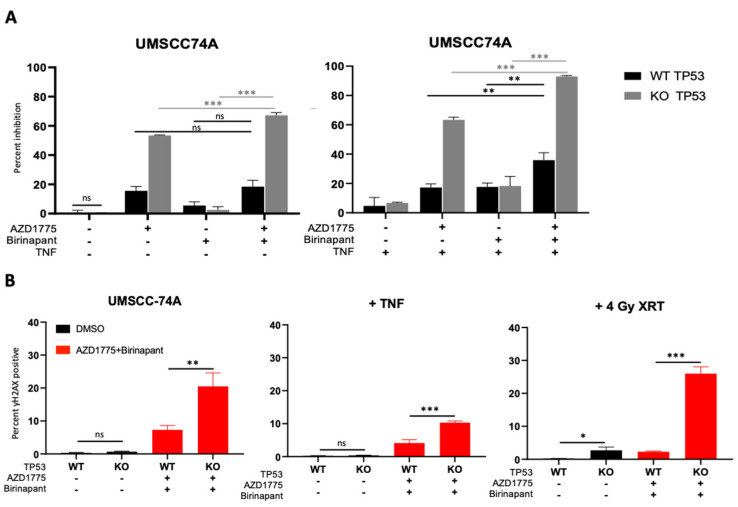
TP53 deficient HNSCC cells are more susceptible to combination therapy cytotoxicity and DNA damage. (**A**) XTT viability analysis in UMSCC74A TP53 WT and KO at 72 h normalized to DMSO control. AZD1775/Birinapant = 500 nM, TNF = 20 ng/mL. n = 3. (**B**) DNA damage according to γ-H2AX positivity on flow cytometry using CRISPR-Cas9 UMSCC-74A p53 KO and parental UMSCC-74A WT. 1 h pretreatment with drugs before 4 Gy radiation then analysis 24 h post treatment. AZD1775/Birinapant = 500 nM, TNF = 20 ng/mL. n = 3. ns = not significant; * *p* < 0.05; ** *p* < 0.01; *** *p* < 0.001.

From our preliminary data in various cell lines, we noticed that the HNSCC cell line that was the least sensitive to combined IAP/WEE1 inhibition was UMSCC-74A, an HPV-negative cell line that retains wild-type TP53 (Figure 1E). TP53 is a cell checkpoint protein that is commonly mutated and inactivated in HPV-negative HNSCC, occurring in 83% of cases, or degraded in HPV-positive HNSCC via the E6 oncogene [11,46]. We therefore sought to identify if TP53 played a role in the response to combined IAP/WEE1 inhibition. To do this, we utilized a CRISPR-Cas9 generated TP53 knockout (KO) version of the wild-type (WT) TP53 UMSCC-74A cell line. Consistent with previous reports, TP53 knockout increases sensitivity to the WEE1 inhibitor AZD1775 and this was further enhanced upon TNFα addition (Figure 5A; [30,47]); however, the response to birinapant was unaffected. Furthermore, TP53 KO enhanced the sensitivity of UMSCC-74A cells to combination treatment when compared with the drug alone; the addition of TNFα further enhanced this effect (Figure 5A). As TP53 plays a role in the DNA damage response, we assessed the role of TP53 on DNA damage induced by the combination treatment. In the absense of treatment, γ-H2AX was only modestly increased in TP53 KO cells in the absence of external stimuli. Following combination treatment, γ-H2AX expression was significantly higher in TP53 KO cells compared with the WT cell line, which was consistent in the presence of TNFα and radiation treatment (Figure 5B). These data suggest that functional TP53 may play a role in modifying the sensitivity to combined IAP/WEE1 inhibition.

### 3.7. Intrinsic TNFα Production Plays a Critical Role in the Response to IAP/WEE1 Inhibition in the Highly Sensitive Cell Line UMSCC-1

**Figure 6:**
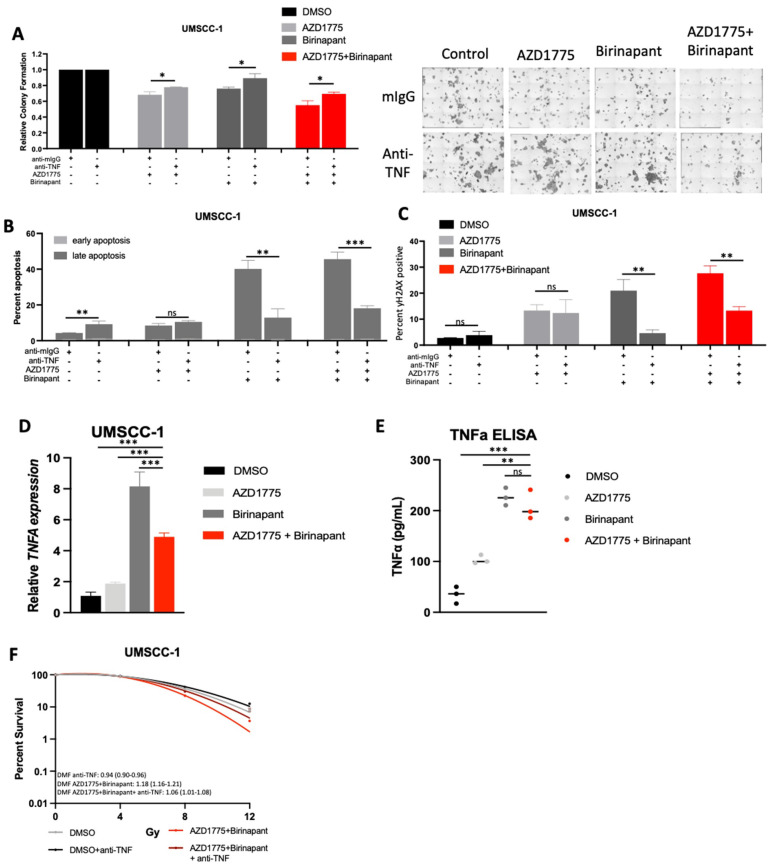
Combination treatment cytotoxicity in UMSCC-1 is at least partially TNF-driven. (**A**) Colony formation after 24 h of treatment with 500 nM AZD1775/Birinapant. Cells were pre-treated with control mouse IgG (1 μg/mL) or anti-TNF (1 μg/mL) for 4 h before treatment. n = 3. (**B**) Annexin apoptosis assay at 24 h with 500 nM AZD1775/Birinapant. Cells were pre-treated with control mouse IgG (1 μg/mL) or anti-TNF (1 μg/mL) for 4 h before treatment. n = 3. (**C**) Flow cytometry analysis of γ-H2AX positivity with 24 h of treatment with 500 nM AZD1775/Birinapant. Cells were pre-treated with control mouse IgG (1 μg/mL) or anti-TNF (1 μg/mL) for 4 h before treatment. n = 3. (**D**) qPCR analysis of TNF following treatment with indicated drug for 24 h, AZD1775/Birinapant = 500 nM, n = 3. U6 was used as a loading control. (**E**) ELISA assay of TNF protein expression following treatment with indicated drug for 24 h, AZD1775/Birinapant = 500 nM, n = 3. (**F**) Clonogenic survival assay with DMF score following 24 h treatment with AZD1775/Birinapant at 500 nM compared to DMSO control +/− anti-TNF (1 μg/mL). Single-dose ionizing radiation was administered 1 h following AZD1775/Birinapant pretreatment in UMSCC-1 (XRT= 0, 4, 8, 12 Gy) and UMSCC-47 (XRT = 0, 2, 4, 6 Gy). ns = not significant; * *p* < 0.05; ** *p* < 0.01; *** *p* < 0.001.

Our data so far have demonstrated that the UMSCC-1 cell line appeared to be uniquely sensitive to combined IAP/WEE1 inhibition, demonstrating increased levels of cytotoxicity, DNA damage, and apoptosis even in the absence of supplemental TNFα or radiation treatment (Supplementary Figure S1). This prompted us to investigate if these effects were mediated by endogenous TNFα, which has previously been shown to mediate some of the cytotoxic effects of both IAP and WEE1 inhibition [30,48]. We therefore performed experiments using an anti- TNFα neutralizing antibody to determine whether intrinsic production of TNFα was contributing to the effects observed. We found that after pre-treatment with an anti-TNFα antibody, there is a significant restoration of colony formation in UMSCC-1 treated with either birinapant, AZD1775 or the combination treatment (Figure 6A). Furthermore, addition of the anti-TNFα antibody significantly reduced the apoptosis and DNA damage induced by birinapant and the combination (Figure 6B,C), suggesting that UMSCC-1 sensitivity to combination treatment in the absence of external TNFα supplementation relies on intrinsic TNFα production. Indeed, we confirmed that the combination treatment induced significant levels of TNFA mRNA expression and protein secretion, primarily driven by IAP inhibition (Figure 6D,E). Finally, treatment with the anti-TNFα antibody reversed the enhanced radio sensitization upon combination treatment in UMSCC-1 cells (Figure 6F). Altogether, these data suggest that the cytotoxicity and radio-sensitization observed upon combined IAP/WEE1 inhibition in UMSCC-1 cells is primarily mediated by induced TNFα production and signaling.

## 4. Discussion

The therapeutic landscape for treatment of head and neck squamous cell carcinoma still relies on surgery and radiotherapy in combination with chemotherapeutics and immunotherapy, particularly for advanced cases [49]. Several novel small molecule therapies are under clinical investigation with the goal of improving radiation treatment efficacy, targeting nonresponsive cancer types, and decreasing toxicities associated with current regimens [22,50,51,52]. Combination therapy with agents that individually or synergistically induce cancer cell death represents an avenue for enhancing efficacy across a wide range of cancers with mutations affecting different cell death pathways, while limiting treatment-associated toxicity [53]. As such, protein ubiquitination and the DNA damage response represent promising targets for novel therapeutic combinations [54,55]. This study investigates a previously unreported combination of the cIAP and WEE1 inhibitor drug classes, which target pathways known to be dysregulated in and critical for cancer cell survival and progression [52,56]. Both drug classes have shown to induce cancer cell death in vitro and in vivo across a panel of HSNCC cell lines [30,48].

Previous work has shown that both IAP and WEE1 inhibitors can enhance cell death induced by both TNFα and ionizing radiation [16,30]. Notably, both cIAP and WEE1 inhibitor drug classes are modulators of the TNFα-NFκB pathway, which regulates proliferation, cell death, and tumor promoting inflammation in HNSCC [57]. While TNFα has long been implicated as an anti-cancer agent, it has been difficult to utilize for treatment since with systemic therapy there is near universal treatment related toxicity and opposing downstream effects which promote resistance [58]. While TNFα is a stimulator of both intrinsic and extrinsic caspase-dependent apoptosis and necroptosis, its effects are often mitigated by downstream activation of RELA/NFκB targets that mediate resistance to cell death [57]. Our data demonstrate that IAP and WEE1 inhibitors increase TNFα-associated cell death [7,8,9,10]. Notably, cIAP proteins increase TNFα-associated cell death, but also increase NFκB signaling, potentially through the autoinduction of TNFα and activation of the non-canonical TNFα pathway as previously reported [13]. Our previous data demonstrated that the WEE1 inhibitor AZD1775 inhibits NFκB activity, and TNFa-induced expression of pro-survival proteins, while also enhancing DNA damage and the potential for mitotic cell death. The combined inhibition of IAP and WEE1 proteins may primarily drive TNFα-induced cell death via the enhanced induction of TNFα, the inhibition of NFκB-induced pro-survival proteins, and the induction of DNA damage.

Our data demonstrate the susceptibility of a panel of HNSCC cancer cell lines with different mutational profiles and HPV status to combined inhibition of IAP and WEE1 in the presence of TNFα, an inflammatory cytokine produced in the tumor microenvironment by immune cells, stomal, and tumor cells when induced by ionizing radiotherapy [6,59]. Multiple HNSCC cell lines showed sensitivity to the combination in the presence of TNFα, especially in cancer cell lines harboring mutations or inactivation of *TP53*. In vitro cytotoxicity of the combination was limited in non-malignant oral keratinocytes and in HNSCC cells expressing wild-type *TP53*. Interestingly, TP53 depletion in the *TP53* WT UMSCC-74A cell line sensitized them to TNFα and combination treatment, supporting the importance of the G2/M checkpoint in sensitivity to WEE1 inhibition. Therefore, *TP53* status may function as a biomarker to predict sensitivity to this combination treatment.

The mechanism of cell death induced from the combination treatment was found to be multifactorial, which supports the importance of the cell cycle and TNFα-NFκB pathway in regulating cell death outcomes. The differences in the primary mechanisms of cell death have been reported to correlate with the expression of key cell death response proteins, including *FADD*, *BIRC2*, and *CASP8*, and may help explain some of the differential sensitivities to the two drugs and combination in different cell lines [16]. In particular, the DNA damage induced by IAP/WEE1 inhibition is enhanced by the presence of TNFα and is likely an important component for the radio-sensitization effects observed. In line with this observation, pre-treatment with the anti-TNFα antibody significantly attenuated the enhanced radio-sensitivity upon combination treatment.

Our data also suggest that the observed sensitization to TNFα-induced cytotoxicity after IAP/WEE1 inhibition may be due to the effects on the IKK-NFκB pathway. We previously demonstrated that WEE1 inhibition impairs the canonical NFκB pathway and thereby attenuates both pro-survival gene expression, sensitizing HNSCC cells to TNFα-induced cell death [30]. Furthermore, we previously reported that birinapant similarly sensitizes HNSCC cells to TNFα-induced cell death, and other groups have demonstrated that birinapant can attenuate the canonical NFκB pathway [16,48,60]. TNFα is present in tumor tissue and systemic anti-TNFα abrogates the effects of radiation in vivo. Thus, the production of TNFα by tumor or other cells in the microenvironment may, therefore, be an indicator and requirement for responsiveness to IAP inhibitor therapy, which relies on TNFα activation for the induction of apoptosis, necroptosis, and DNA-damage-associated cell death.

The present study has several limitations. Our data are derived from in vitro models, whereas an in vivo model will be required to determine the toxicity and kinetics of combination therapy with IAP inhibitors and WEE1 inhibitors in the presence of radiation. Furthermore, our panel of cell lines had differential susceptibility to the individual agents and combination treatment. Additional work is required to differentiate tumor cell characteristics that predict synergistic cytotoxicity in response to combination treatment. Finally, our in vitro radiation studies utilized single-dose ionizing radiation; however the dosage and temporal relationship to small molecule inhibitor treatment requires further investigation and optimization.

In general, this study provides preclinical data supporting the future investigation of combination therapy with IAP and WEE1 inhibitors to target *TP53* mutated HNSCC. Additional work using novel 3D organotypic cultures of HNSCC would strengthen our preclinical data, as would the use of murine models to determine in vivo activity and identify additional predictive markers of HNSCC that will have clinically significant responses [61,62].

## 5. Conclusions

Head and neck squamous cell carcinoma (HNSCC) remains a prevalent diagnosis that requires further investigation to identify novel regimens to treat aggressive tumor types and reduce the toxicity of current treatment options. Tumor necrosis factor-α (TNFα) is a key mediator of cytotoxicity that is frequently dysregulated in carcinoma and can be induced by a variety of chemo-, radio-, and immunotherapies. Previous studies have demonstrated the ability of two novel small molecule inhibitors, the IAP inhibitor birinapant and the WEE1 inhibitor AZD1775, to sensitize HNSCC to TNFα dependent cell killing in vitro and radiotherapy in vivo. Our results demonstrate that combination treatment with birinapant and AZD1775 impairs proliferation and induces cytotoxicity in both HPV-negative and HPV-positive HNSCC cell lines, in the presence of both TNFα and radiation treatment in vitro. The mechanism of action in multifactorial and correlates with induction of apoptosis/necroptosis, cell cycle dysregulation, and DNA damage. These results support the additional exploration of IAP and WEE1 inhibitor combinations for the treatment of HNSCC in vivo pre-clinical models.

## Data Availability

The data presented in this study are available in the article.

## Supplementary Materials

The following supporting information can be downloaded at: https://www.mdpi.com/article/10.3390/cancers15041029/s1, Figure S1: Combined treatment with AZD1775 and Birinapant has reduced combinatorial cytotoxic activity in the absence TNF. Figure S2. The alternate IAP inhibitor ASTX660 also produces combinatorial effects with AZD1775 treatment in HNSCC cells. Figure S3. Validation of downstream target inhibition. Figure S4. Cell cycle and cell death effects of combination treatment in the absence of TNF. Figure S5. Combination treatment reduces NFkB activity and IKK/RELA phosphorylation in HNSCC cells.

## References

[1] Sacco A.G., Cohen E.E. (2015). Current Treatment Options for Recurrent or Metastatic Head and Neck Squamous Cell Carcinoma. J. Clin. Oncol..

[2] Wu Q., Wang M., Liu Y., Wang X., Li Y., Hu X., Qiu Y., Liang W., Wei Y., Zhong Y. (2021). HPV Positive Status Is a Favorable Prognostic Factor in Non-Nasopharyngeal Head and Neck Squamous Cell Carcinoma Patients: A Retrospective Study From the Surveillance, Epidemiology, and End Results Database. Front. Oncol..

[3] Li H., Torabi S., Yarbrough W.G., Mehra S., Osborn H.A., Judson B. (2018). Association of Human Papillomavirus Status at Head and Neck Carcinoma Subsites With Overall Survival. JAMA Otolaryngol.–Head Neck Surg..

[4] Chitsike L., Duerksen-Hughes P.J. (2021). Targeted Therapy as a Potential De-Escalation Strategy in Locally Advanced HPV-Associated Oropharyngeal Cancer: A Literature Review. Front. Oncol..

[5] Stuelten C.H., Byfield S.D., Arany P.R., Karpova T.S. (2005). Breast cancer cells induce stromal fibroblasts to express MMP-9 via secretion of TNF-alpha and TGF-beta. J. Cell Sci..

[6] Laha D., Grant R., Mishra P., Nilubol N. (2021). The Role of Tumor Necrosis Factor in Manipulating the Immunological Response of Tumor Microenvironment. Front. Immunol..

[7] Wang X., Lin Y. (2008). Tumor necrosis factor and cancer, buddies or foes?. Acta Pharm. Sin..

[8] Wang C.-Y., Mayo M.W., Baldwin A.S. (1996). TNF- and Cancer Therapy-Induced Apoptosis: Potentiation by Inhibition of NF-kB. Science.

[9] Verzella D., Pescatore A., Capece D., Vecchiotti D., Ursini M.V., Franzoso G., Alesse E., Zazzeroni F. (2020). Life, death, and autophagy in cancer: NF-κB turns up everywhere. Cell Death Dis..

[10] Xia Y., Shen S., Verma I.M. (2014). NF-κB, an active player in human cancers. Cancer Immunol. Res..

[11] Lawrence M.S. (2015). Comprehensive genomic characterization of head and neck squamous cell carcinomas. Nature.

[12] Mayo M.W., Wang C.Y., Cogswell P.C., Rogers-Graham K.S., Lowe S.W., Der C.J., Baldwin A.S. (1997). Requirement of NF-kB Activation to Suppress p53-Independent Apoptosis Induced by Oncogenic Ras. Science.

[13] Tchoghandjian A., Jennewein C., Eckhardt I., Rajalingam K., Fulda S. (2013). Identification of non-canonical NF-κB signaling as a critical mediator of Smac mimetic-stimulated migration and invasion of glioblastoma cells. Cell Death Dis..

[14] Fulda S. (2014). Molecular pathways: Targeting inhibitor of apoptosis proteins in cancer--from molecular mechanism to therapeutic application. Clin. Cancer Res..

[15] Brands R.C., Scheurer M.J., Hartmann S., Seher A., Kübler A., Müller-Richter U. (2018). Apoptosis-sensitizing activity of birinapant in head and neck squamous cell carcinoma cell lines. Oncol. Lett..

[16] Eytan D.F., Snow G.E., Carlson S., Derakhshan A., Saleh A., Schiltz S., Cheng H., Mohan S., Cornelius S., Coupar J. (2016). SMAC Mimetic Birinapant plus Radiation Eradicates Human Head and Neck Cancers with Genomic Amplifications of Cell Death Genes FADD and BIRC2. Cancer Res..

[17] Xiao R., An Y., Ye W., Derakhshan A., Cheng H., Yang X., Allen C., Chen Z., Schmitt N.C., Van Waes C. (2019). Dual Antagonist of cIAP/XIAP ASTX660 Sensitizes HPV(−) and HPV(+) Head and Neck Cancers to TNFα, TRAIL, and Radiation Therapy. Clin. Cancer Res..

[18] Xiao R., Allen C.T., Tran L., Patel P., Park S.J., Chen Z., Van Waes C., Schmitt N.C. (2018). Antagonist of cIAP1/2 and XIAP enhances anti-tumor immunity when combined with radiation and PD-1 blockade in a syngeneic model of head and neck cancer. OncoImmunology.

[19] Ye W., Gunti S., Allen C.T., Hong Y., Clavijo P.E., Van Waes C., Schmitt N.C. (2020). ASTX660, an antagonist of cIAP1/2 and XIAP, increases antigen processing machinery and can enhance radiation-induced immunogenic cell death in preclinical models of head and neck cancer. Oncoimmunology.

[20] Sun X.-S., Tao Y., Le Tourneau C., Pointreau Y., Sire C., Kaminsky M.-C., Coutte A., Alfonsi M., Boisselier P., Martin L. (2020). Debio 1143 and high-dose cisplatin chemoradiotherapy in high-risk locoregionally advanced squamous cell carcinoma of the head and neck: A double-blind, multicentre, randomised, phase 2 study. Lancet Oncol..

[21] Schoenfeld J., Cohen E., Nutting C., Licitra L., Burtness B., Omar M., Bouisset F., Nauwelaerts H., Urfer Y., Zanna C. (2022). Trilynx: A Phase 3 Trial of Xevinapant and Concurrent Chemoradiotherapy (CRT) for Locally Advanced Head and Neck Cancer. Int. J. Radiat. Oncol. Biol. Phys..

[22] Bourhis J., Burtness B., Licitra L.F., Nutting C., Schoenfeld J.D., Sarkouh R.A., Bouisset F., Nauwelaerts H., Urfer Y., Zanna C. (2021). TrilynX: A phase 3 trial of xevinapant and concurrent chemoradiation for locally advanced head and neck cancer. J. Clin. Oncol..

[23] Bourhis J., Burtness B., Licitra L.F., Nutting C., Schoenfeld J.D., Omar M., Bouisset F., Nauwelaerts H., Urfer Y., Zanna C. (2022). Xevinapant or placebo plus chemoradiotherapy in locally advanced squamous cell carcinoma of the head and neck: TrilynX phase III study design. Future Oncol..

[24] Debiopharm, FDA Grants Breakthrough Therapy Designation for Debiopharm’s Novel Chemo-Radio Sensitizer Debio 1143 for Front-Line Treatment of Head & Neck Cancer February 27, 2020: Lausanne, Switzerland. https://www.debiopharm.com/drug-development/press-releases/fda-grants-breakthrough-therapy-designation-for-debiopharms-novel-chemo-radio-sensitizer-debio-1143-for-front-line-treatment-of-head-neck-cancer/.

[25] Bukhari A.B., Chan G.K., Gamper A.M. (2022). Targeting the DNA Damage Response for Cancer Therapy by Inhibiting the Kinase Wee1. Front. Oncol..

[26] Ghelli Luserna di Rorà A., Cerchione C., Martinelli G., Simonetti G. (2020). A WEE1 family business: Regulation of mitosis, cancer progression, and therapeutic target. J. Hematol. Oncol..

[27] Bi S., Wei Q., Zhao Z., Chen L., Wang C., Xie S. (2019). Wee1 Inhibitor AZD1775 Effectively Inhibits the Malignant Phenotypes of Esophageal Squamous Cell Carcinoma In Vitro and In Vivo. Front. Pharmacol..

[28] Oza A.M., Estevez-Diz M.D.P., Grischke E.-M., Hall M., Marmé F., Provencher D.M., Uyar D.S., Weberpals J.I., Wenham R.M., Laing N. (2020). A Biomarker-enriched, Randomized Phase II Trial of Adavosertib (AZD1775) Plus Paclitaxel and Carboplatin for Women with Platinum-sensitive TP53-mutant Ovarian Cancer. Clin. Cancer Res..

[29] Cole K.A., Pal S., Kudgus R.A., Ijaz H., Liu X., Minard C.G., Pawel B.R., Maris J.M., Haas-Kogan D.A., Voss S.D. (2020). Phase I Clinical Trial of the Wee1 Inhibitor Adavosertib (AZD1775) with Irinotecan in Children with Relapsed Solid Tumors: A COG Phase I Consortium Report (ADVL1312). Clin. Cancer Res..

[30] Hu Z., Viswanathan R., Cheng H., Chen J., Yang X., Huynh A., Clavijo P., An Y., Robbins Y., Silvin C. (2022). Inhibiting WEE1 and IKK-RELA Crosstalk Overcomes TNFα Resistance in Head and Neck Cancers. Mol. Cancer Res..

[31] Cheng H., Yang X., Si H., Saleh A.D., Xiao W., Coupar J., Gollin S.M., Ferris R.L., Issaeva N., Yarbrough W.G. (2018). Genomic and Transcriptomic Characterization Links Cell Lines with Aggressive Head and Neck Cancers. Cell Rep..

[32] Mita M.M., LoRusso P.M., Papadopoulos K.P., Gordon M.S., Mita A.C., Ferraldeschi R., Keer H., Oganesian A., Su X.Y., Jueliger S. (2020). A Phase I Study of ASTX660, an Antagonist of Inhibitors of Apoptosis Proteins, in Adults with Advanced Cancers or Lymphoma. Clin. Cancer Res..

[33] Bliss C.I. (1939). The Toxicity of Poisons Applied Jointly. Ann. Appl. Biol..

[34] Plesca D., Mazumder S., Almasan A. (2008). DNA damage response and apoptosis. Methods Enzym..

[35] Patel P., Sun L., Robbins Y., Clavijo P.E., Friedman J., Silvin C., Van Waes C., Cook J., Mitchell J., Allen C. (2019). Enhancing direct cytotoxicity and response to immune checkpoint blockade following ionizing radiation with Wee1 kinase inhibition. Oncoimmunology.

[36] Wright G., Golubeva V., Rix L.L.R., Berndt N., Luo Y., Ward G.A., Gray J.E., Schonbrunn E., Lawrence H.R., Monteiro A.N. (2017). Dual Targeting of WEE1 and PLK1 by AZD1775 Elicits Single Agent Cellular Anticancer Activity. ACS Chem. Biol..

[37] Luo J.L., Kamata H., Karin M. (2005). IKK/NF-kappaB signaling: Balancing life and death--a new approach to cancer therapy. J. Clin. Invest..

[38] Varfolomeev E., Blankenship J.W., Wayson S.M., Fedorova A.V., Kayagaki N., Garg P., Zobel K., Dynek J.N., Elliott L.O., Wallweber H.J.A. (2007). IAP Antagonists Induce Autoubiquitination of c-IAPs, NF-κB Activation, and TNFα-Dependent Apoptosis. Cell.

[39] Vince J.E., Wong W.W.-L., Khan N., Feltham R., Chau D., Ahmed A.U., Benetatos C.A., Chunduru S.K., Condon S.M., McKinlay M. (2007). IAP Antagonists Target cIAP1 to Induce TNFα-Dependent Apoptosis. Cell.

[40] Grénman R., Carey T.E., McClatchey K.D., Wagner J.G., Pekkola-Heino K., Ms D.R.S., Wolf G.T., Lacivita L.P., Ho L., Baker S.R. (1991). In vitro radiation resistance among cell lines established from patients with squamous cell carcinoma of the head and neck. Cancer.

[41] Kinner A., Wu W., Staudt C., Iliakis G. (2008). Gamma-H2AX in recognition and signaling of DNA double-strand breaks in the context of chromatin. Nucleic Acids Res..

[42] Spary L., Al-Taei S., Salimu J., Cook A.D., Ager A., Watson H.A., Clayton A., Staffurth J., Mason M.D., Tabi Z. (2014). Enhancement of T Cell Responses as a Result of Synergy between Lower Doses of Radiation and T Cell Stimulation. J. Immunol..

[43] Zhou Z., Zhao J., Hu K., Hou X., Sun X., Pan X., Wang X., Li N., Yang Z., Zhang F. (2021). Single High-Dose Radiation Enhances Dendritic Cell Homing and T Cell Priming by Promoting Reactive Oxygen Species-Induced Cytoskeletal Reorganization. Int. J. Radiat. Oncol. Biol. Phys..

[44] Ziemann F., Arenz A., Preising S., Wittekindt C., Klussmann J.P., Engenhart-Cabillic R., Wittig A. (2015). Increased sensitivity of HPV-positive head and neck cancer cell lines to x-irradiation ± Cisplatin due to decreased expression of E6 and E7 oncoproteins and enhanced apoptosis. Am. J. Cancer Res..

[45] Kimple R.J., Smith M.A., Blitzer G.C., Torres A.D., Martin J.A., Yang R.Z., Peet C.R., Lorenz L.D., Nickel K.P., Klingelhutz A.J. (2013). Enhanced radiation sensitivity in HPV-positive head and neck cancer. Cancer Res..

[46] Thomas M., Pim D., Banks L. (1999). The role of the E6-p53 interaction in the molecular pathogenesis of HPV. Oncogene.

[47] Diab A., Gem H., Swanger J., Kim H.Y., Smith K., Zou G., Raju S., Kao M., Fitzgibbon M., Loeb K.R. (2020). FOXM1 drives HPV+ HNSCC sensitivity to WEE1 inhibition. Proc. Natl. Acad. Sci. USA.

[48] Eytan D.F., Snow G.E., Carlson S.G., Schiltz S., Chen Z., Van Waes C. (2015). Combination effects of SMAC mimetic birinapant with TNFα, TRAIL, and docetaxel in preclinical models of HNSCC. Laryngoscope.

[49] Johnson D.E., Burtness B., Leemans C.R., Lui V.W., Bauman J.E., Grandis J.R. (2020). Head and neck squamous cell carcinoma. Nat. Rev. Dis. Prim..

[50] Derakhshan A., Chen Z., Van Waes C. (2017). Therapeutic Small Molecules Target Inhibitor of Apoptosis Proteins in Cancers with Deregulation of Extrinsic and Intrinsic Cell Death Pathways. Clin. Cancer Res..

[51] Marquard F.E., Jücker M. (2020). PI3K/AKT/mTOR signaling as a molecular target in head and neck cancer. Biochem. Pharm..

[52] van Harten A.M., de Boer D.V., Martens-de Kemp S.R., Buijze M., Ganzevles S.H., Hunter K.D., Leemans C.R., van Beusechem V.W., Wolthuis R.M., de Menezes R.X. (2020). Chemopreventive targeted treatment of head and neck precancer by Wee1 inhibition. Sci. Rep..

[53] Mokhtari R.B., Homayouni T.S., Baluch N., Morgatskaya E., Kumar S., Das B., Yeger H. (2017). Combination therapy in combating cancer. Oncotarget.

[54] Morgan E.L., Chen Z., Van Waes C. (2020). Regulation of NFκB Signalling by Ubiquitination: A Potential Therapeutic Target in Head and Neck Squamous Cell Carcinoma?. Cancers.

[55] Hutchinson M.-K.N.D., Mierzwa M., D’Silva N.J. (2020). Radiation resistance in head and neck squamous cell carcinoma: Dire need for an appropriate sensitizer. Oncogene.

[56] Tanimoto T., Tsuda H., Imazeki N., Ohno Y., Imoto I., Inazawa J., Matsubara O. (2005). Nuclear expression of cIAP-1, an apoptosis inhibiting protein, predicts lymph node metastasis and poor patient prognosis in head and neck squamous cell carcinomas. Cancer Lett..

[57] Hayden M.S., Ghosh S. (2014). Regulation of NF-κB by TNF family cytokines. Semin. Immunol..

[58] Roberts N.J., Zhou S., Diaz L.A., Holdhoff M. (2011). Systemic use of tumor necrosis factor alpha as an anticancer agent. Oncotarget.

[59] Citrin D.E., Hitchcock Y.J., Chung E.J., Frandsen J., Urick M.E., Shield W., Gaffney D. (2012). Determination of cytokine protein levels in oral secretions in patients undergoing radiotherapy for head and neck malignancies. Radiat. Oncol..

[60] Benetatos C.A., Mitsuuchi Y., Burns J.M., Neiman E.M., Condon S.M., Yu G., Seipel M.E., Kapoor G.S., LaPorte M.G., Rippin S.R. (2014). Birinapant (TL32711), a bivalent SMAC mimetic, targets TRAF2-associated cIAPs, abrogates TNF-induced NF-κB activation, and is active in patient-derived xenograft models. Mol. Cancer Ther..

[61] Apu E.H., Akram S.U., Rissanen J., Wan H., Salo T. (2018). Desmoglein 3–Influence on oral carcinoma cell migration and invasion. Exp. Cell Res..

[62] Engelmann L., Thierauf J., Laureano N.K., Stark H.-J., Prigge E.-S., Horn D., Freier K., Grabe N., Rong C., Federspil P. (2020). Organotypic co-cultures as a novel 3D model for head and neck squamous cell carcinoma. Cancers.

