# Supplementary material for: Combined Inhibition of IAPs and WEE1 Enhances TNFα- and Radiation-Induced Cell Death in Head and Neck Squamous Carcinoma
Source: Cancers (Basel). 2023 Feb 6;15(4):1029. doi: 10.3390/cancers15041029 (PMC9954698; doi:10.3390/cancers15041029)
Supplement: Supplementary file 1 [file cancers-15-01029-s001.zip › cancers-2206671-supplementary.pdf]

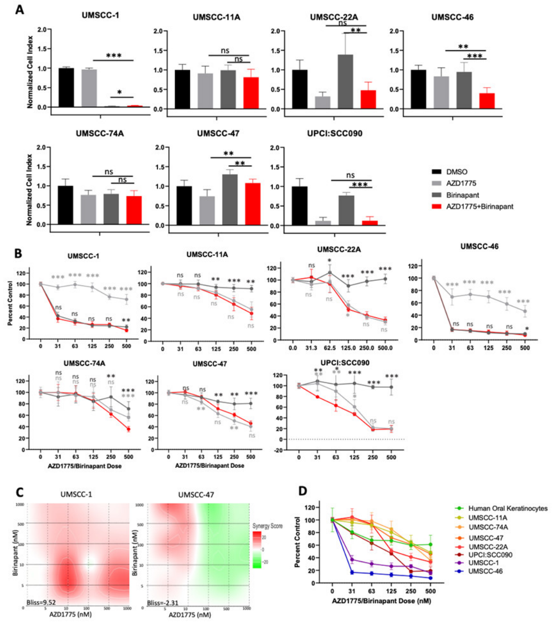

Supplementary Figure S1. Combined treatment with AZD1775 and Birinapant has reduced combinatorial cytotoxic activity in the absence TNF.

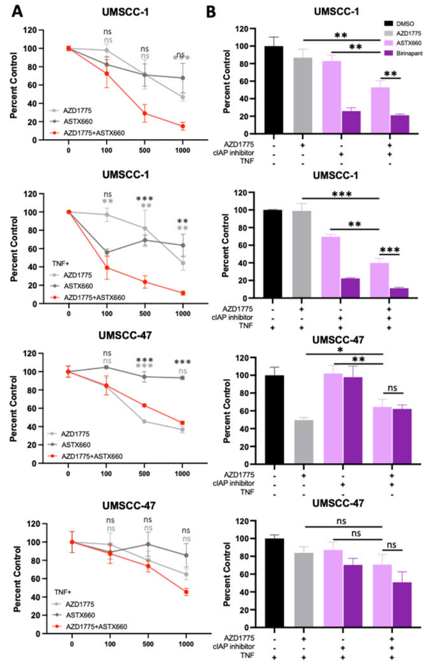

Supplementary Figure S2. The alternate IAP inhibitor ASTX660 also produces combinatorial effects with AZD1775 treatment in HNSCC cells.

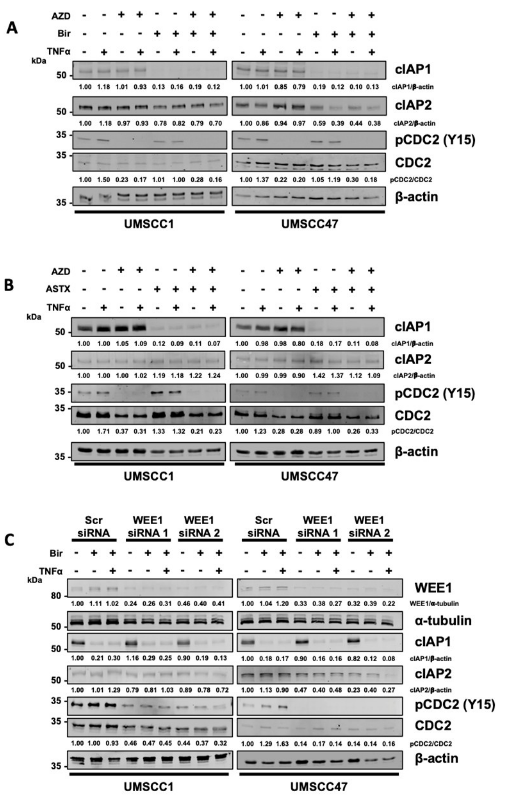

Supplementary Figure S3. Validation of downstream target inhibition.

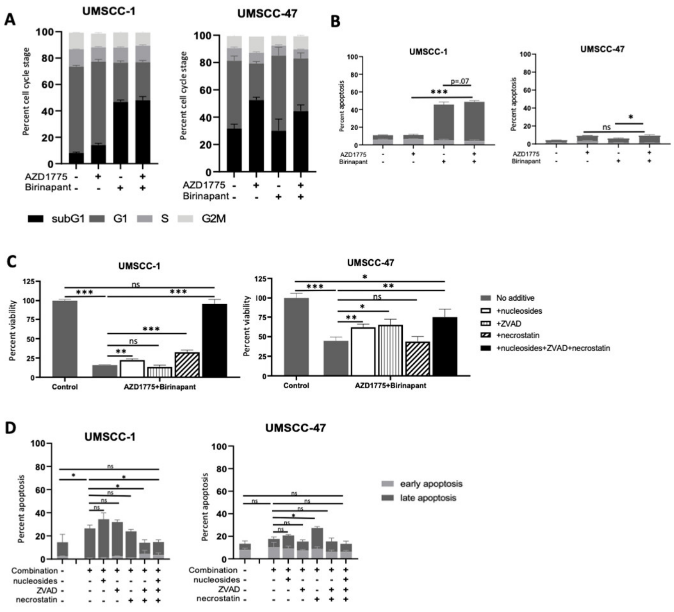

Supplementary Figure S4. Cell cycle and cell death effects of combination treatment in the absence of TNF.

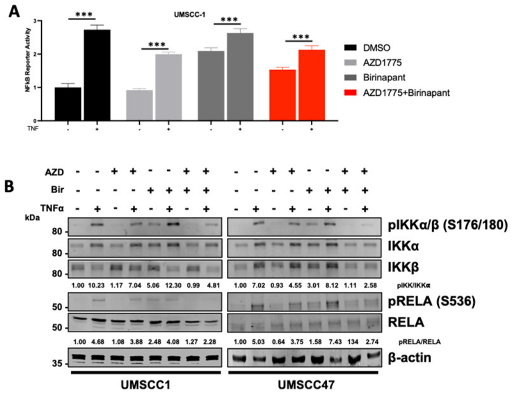

Supplementary Figure S5. Combination treatment reduces NFkB activity and IKK/RELA phosphorylation in HNSCC cells.
